# Awareness of Colorectal Cancer Risk Factors in Palestine: Where Do We Stand?

**DOI:** 10.1200/GO.22.00070

**Published:** 2022-06-13

**Authors:** Mohamedraed Elshami, Mohammad F. Dwikat, Ibrahim Al-Slaibi, Mohammed Alser, Balqees M. Mohamad, Wejdan S. Isleem, Adela Shurrab, Bashar Yaghi, Yahya Ayyash Qabaja, Shoruq A. Naji, Fatima K. Hmdan, Mohammed M. Ayyad, Raneen R. Sweity, Remah T. Jneed, Khayria A. Assaf, Maram E. Albandak, Mohammed M. Hmaid, Iyas I. Awwad, Belal K. Alhabil, Marah N. Alarda, Amani S. Alsattari, Moumen S. Aboyousef, Omar A. Aljbour, Rinad AlSharif, Christy T. Giacaman, Ali Y. Alnaga, Ranin M. Abu Nemer, Nada M. Almadhoun, Sondos M. Skaik, Bettina Bottcher, Nasser Abu-El-Noor

**Affiliations:** ^1^ Division of Surgical Oncology, Department of Surgery, University Hospitals Cleveland Medical Center, Cleveland, OH; ^2^ Ministry of Health, Gaza, Palestine; ^3^ Faculty of Medicine, An-Najah National University, Nablus, Palestine; ^4^ Almakassed Hospital, Jerusalem, Palestine; ^5^ Beit Jala Governmental Hospital (Al-Hussein), Bethlehem, Palestine; ^6^ Faculty of Medicine, Islamic University of Gaza, Gaza, Palestine; ^7^ Palestine Medical Complex, Khanyounis, Palestine; ^8^ Faculty of Medicine, Al-Quds University, Jerusalem, Palestine; ^9^ Faculty of Pharmacy, Al-Azhar University of Gaza, Gaza, Palestine; ^10^ Faculty of Dentistry, Arab American University, Palestine, Jenin; ^11^ Faculty of Nursing and Health Sciences, Bethlehem University, Bethlehem, Palestine; ^12^ Faculty of Allied Medical Sciences, Arab American University, Jenin, Palestine; ^13^ Faculty of Medicine, Al-Azhar University, Gaza, Palestine; ^14^ Faculty of Medicine, Al-Quds Abu Dis University Al-Azhar Branch of Gaza, Gaza, Palestine; ^15^ Faculty of Nursing, Islamic University of Gaza, Gaza, Palestine

## Abstract

To assess the public awareness level of colorectal cancer (CRC) risk factors in Palestine and identify factors associated with the good awareness level.

**MATERIALS AND METHODS:**

Adult Palestinians were recruited using convenience sampling from hospitals, primary health care centers, and public locations in 11 governorates. The recognition of 11 CRC risk factors was evaluated using a translated-into-Arabic version of the validated bowel cancer awareness measure. Participants were given one point for each correctly recognized risk factor. The awareness level was determined by the number of CRC risk factors recognized: poor (0-3), fair (4-7), and good awareness (8-11).

**RESULTS:**

A total of 4,877 participants, of 5,254 approached, completed the questionnaire (response rate = 92.3%). The final analysis included 4,623 questionnaires, 2,700 from the West Bank and Jerusalem (WBJ) and 1,923 from the Gaza Strip. Participants from the WBJ were older, gained higher monthly income, and had more chronic diseases than participants from the Gaza Strip. The most recognized modifiable CRC risk factor was not doing 30 minutes of moderate physical activity five times a week (n = 3,846, 83.2%), whereas the least recognized was having a diet low in fiber (n = 1,985, 42.9%). The most recognized nonmodifiable CRC risk factor was having a bowel disease (n = 3,320, 71.8%), whereas the least recognized was having diabetes (n = 1,581, 34.2%). Only 1,840 participants (39.8%) demonstrated good awareness of CRC risk factors. Participants from the Gaza Strip were more likely than participants from the WBJ to have good awareness (46.5.0% *v* 35.0%). Female sex, knowing someone with cancer, and completing postsecondary education were all associated with good awareness.

**CONCLUSION:**

Awareness of CRC risk factors was found to be low in Palestine. There is a substantial need to raise awareness of CRC risk factors through educational campaigns and programs.

## INTRODUCTION

Colorectal cancer (CRC) is the third most common malignancy worldwide. CRC was responsible for 1,931,590 new cancer cases (about 10% of all new cases) and 935,173 cancer-related deaths (9.4% of all cancer-related deaths) in 2020.^1^ In Palestine, cancer accounted for 14.1% of total reported deaths and was the third leading cause of death in 2020. CRC is the second most common malignancy with an incidence rate of 13.6 per 100,000 general population in the West Bank and Jerusalem (WBJ) and 11.5 per 100,000 general population in the Gaza Strip.^2,3^ In addition, CRC is the second leading cause of cancer-related deaths (13.9%).^3^

**CONTEXT**

**Key Objective**
Colorectal cancer (CRC) is responsible for a significant number of cancer diagnoses and deaths in Palestine, making it a major public health problem. Therefore, this national study assessed the public awareness of CRC risk factors and examined the sociodemographic factors associated with good awareness.
**Knowledge Generated**
The awareness level of CRC risk factors was relatively low with only 39.8% of participants displaying good awareness. Factors associated with good awareness included living in the West Bank and Jerusalem, completing postsecondary education, and knowing someone with cancer.
**Relevance**
Poor public knowledge of CRC risk factors may play a role in the diagnosis of CRC at advanced stages because of delayed seeking of medical advice, ultimately leading to a lower survival rate. Systematic educational campaigns and programs aiming to promote awareness of CRC are needed and should be tailored to address the knowledge gaps among the public.

CRC risk factors can be classified into modifiable and nonmodifiable risk factors. The modifiable risk factors include physical activity, body mass index, the amount of processed and red meat ingested, the amount of fruits and vegetables as well as fiber in the diet, smoking, and drinking alcohol. On the other hand, the nonmodifiable risk factors include older age, family history, inflammatory bowel disease, and diabetes.^4^

Implementing screening programs has been proven to substantially improve survival and prognosis of patients with CRC.^5,6^ However, previous studies demonstrated that patients' lack of information regarding CRC risk factors may affect their participation in screening programs and therefore may lead to late diagnosis and lower survival rates.^7,8^ Moreover, almost one third of all cancers can be prevented by following a healthy diet, maintaining physical activity, and having a normal body mass index.^9^ This highlights the importance of good awareness of CRC risk factors.

The awareness of CRC risk factors was found to be low in the Gaza Strip.^10^ Nonetheless, there is an unmet need to measure the public awareness across Palestine to establish a baseline for future standardized educational interventions by health authorities and policy makers. Therefore, this study aimed to (1) assess the awareness level of CRC risk factors in Palestine, (2) compare the CRC risk factors' awareness level between the WBJ versus the Gaza Strip, and (3) examine factors associated with good awareness of CRC risk factors.

## MATERIALS AND METHODS

### Study Design and Population

This was a national cross-sectional study conducted between July 2019 and March 2020. There are 16 governorates in Palestine: 11 are located in the WBJ and five in the Gaza Strip. In 2019, the number of adults in Palestine reached approximately 2.6 million. This made up 51.6% of the total Palestinian population (about 5 million).^11^ Therefore, Palestinian adults (≥ 18 years) residing in the WBJ or the Gaza Strip were the target population. Participants were recruited from governmental hospitals, primary health care centers (PHCs), and public spaces across Palestine.

### Sampling Methods

Palestinians in the WBJ and the Gaza Strip receive their health care services in governmental facilities, nongovernmental organizations, the United Nations Relief and Works Agency facilities, and private health care providers. Nevertheless, people rely mostly on governmental hospitals because of the relatively low-cost health insurance, which allows them to use health services at no cost or with low co-payments.^3^ Therefore, convenience sampling was used to recruit participants from 11 governmental hospitals, 12 PHCs, and public spaces in the corresponding 11 governorates (seven in the WBJ and four in the Gaza Strip) of the 16 governorates in Palestine. Public spaces included parks, malls, trade streets, mosques, churches, downtown areas, transportation stations, and others.

### Inclusion and Exclusion Criteria

The inclusion criteria to take part in the study were being an adult (≥ 18 years) Palestinian and visiting one of the included data collection sites. Exclusion criteria were having a citizenship other than Palestinian, visiting oncology departments in PHCs and hospitals at the time of data collection, and studying or working in a health care–related field.

### Data Collection and Measurement Tool

A translated-into-Arabic version of the Bowel Cancer Awareness Measure (BoCAM) was used for data collection. The original BoCAM was developed by University College London and Cancer Research, UK. It is a validated tool for assessing public CRC awareness.^12^ Two bilingual health care professionals translated the questionnaire from English to Arabic, and then it was back translated into English by another two bilingual health care professionals. All these were experts in clinical research and survey design. In addition, five independent specialists in the fields of public health, coloproctology, and gastroenterology reviewed the questionnaire subsequently to ensure content validity and accuracy of translation. After that, a pilot study was conducted (n = 25) to test the clarity of questions in the Arabic BoCAM. The data collected from the pilot study were not included in the final analysis. The questionnaire's internal reliability was assessed using Cronbach's α, which was acceptable with a value of .89.

The questionnaire consisted of two sections. The first section covered the sociodemographic characteristics of participants including age, sex, marital status, level of education, employment status, monthly income, place of residence, having a chronic health condition, following a vegetarian diet, and knowing someone with cancer. The second section assessed the participant's recognition of 11 CRC risk factors on the basis of a five-point Likert scale (strongly disagree, disagree, not sure, agree, and strongly agree). Of the 11 CRC risk factors, 10 were adopted from the original BoCAM,^13^ and smoking cigarettes was added as it was deemed important given its high prevalence in the Palestinian community.^14^

Participants were invited for a face-to-face interview to complete the questionnaire. The Kobo Toolbox, an easy-to-use and secure tool that can be accessed via smartphones, was used to collect data.^15^ Before starting data collection, data collectors received training to learn how to use the Kobo Toolbox and how to approach participants and facilitate their completion of the questionnaire.

### Ethics Approval and Consent to Participate

Before data collection, ethical approval had been sought from the Research Ethics Committee at the Islamic University of Gaza, the Human Resources Development department at the Palestinian Ministry of Health, and the Helsinki Committee in the Gaza Strip. In addition, the participants had a thorough explanation about the study including its purpose and objectives with the focus that their participation is completely voluntary. Written informed consent was obtained from each participant before starting the questionnaire, and data were collected anonymously.

### Statistical Analysis

The American Cancer Society recommends starting CRC screening at age 45 years for people at average risk of developing CRC.^16^ Therefore, the continuous variable of age was classified into two categories using this cutoff: 18-44 years and ≥ 45 years. In Palestine, the minimum wage is 1,450 Israeli new shekel (NIS) (about $450 US dollars),^17^ and therefore, monthly income was classified into two categories using that as a cutoff: < 1,450 NIS and ≥ 1,450 NIS.

The median [interquartile range] was used to summarize continuous, non-normally distributed variables, and the Kruskal-Wallis test was used to perform a baseline comparison between participants from the WBJ versus the Gaza Strip. Frequencies and percentages were used to summarize categorical variables, and Pearson's chi-square test was used for baseline comparisons.

The prompt recognition of each CRC risk factor was assessed using a question on the basis of a five-point Likert scale, with strongly agree or agree deemed as a correct answer and strongly disagree, disagree, or not sure deemed as an incorrect answer. CRC risk factors were further classified into two main categories: (1) modifiable and (2) nonmodifiable risk factors. Recognizing each CRC risk factor was described using frequencies and percentages with comparisons performed by Pearson's chi-square test. Bivariable and multivariable logistic regression analyses were then used to examine the association between the recognition of each CRC risk factor and participant characteristics. The multivariable analyses adjusted for age group, sex, educational level, occupation, monthly income, place of residency, marital status, having a chronic disease, following a vegetarian diet, knowing someone with cancer, and site of data collection. This model was determined a priori on the basis of previous studies.^10,18-22^ Results of the bivariable analyses are provided in Appendix Tables A1 and A2.

To evaluate the awareness level among study participants about CRC risk factors, a scoring system was used. Similar scoring systems were also used in previous studies.^22-27^ The total score (ranging from 0 to 11) was calculated and classified into three categories on the basis of the number of CRC risk factors recognized: poor (0-3), fair (4-7), and good awareness (8-11). The awareness level of CRC risk factors among participants from the WBJ versus the Gaza Strip was compared using Pearson's chi-square test. This was followed by running bivariable and multivariable logistic regression analyses to test the association between having good awareness of CRC risk factors and participant characteristics.

Complete case analysis was used to handle missing data as they occurred completely at random. Data were analyzed using Stata software version 16.0 (StataCorp, College Station, TX).

## RESULTS

### Participant Characteristics

A total of 4,877 participants, of 5,254 approached, completed the questionnaire (response rate = 92.3%). The final analysis included 4,623 questionnaires (254 excluded: 44 did not meet the inclusion criteria and 210 had missing data): 2,700 from the WBJ and 1,923 from the Gaza Strip. Among all participants, the median age [interquartile range] was 31.0 years [24.0-43.0] and 1,879 (40.6%) were males (Table 1). Participants from the WBJ were older, gained higher monthly income, and had more chronic diseases than participants from the Gaza Strip.

**TABLE 1:**
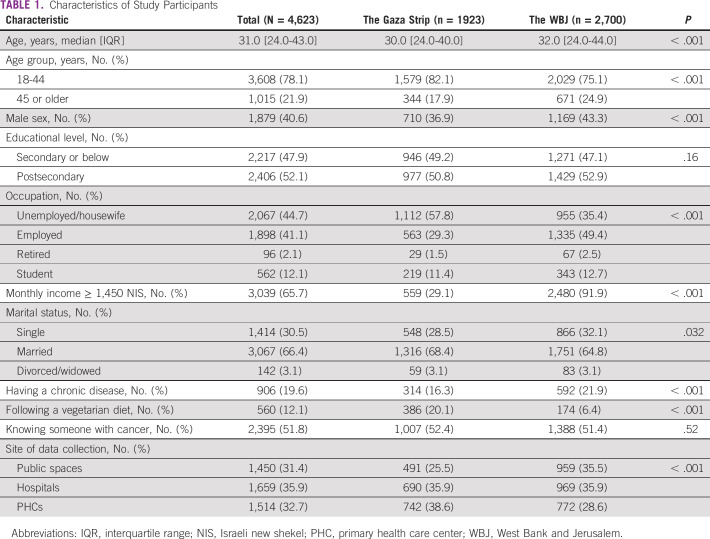
Characteristics of Study Participants

### Recognition of CRC Risk Factors

The most recognized modifiable CRC risk factor was not doing 30 minutes of moderate physical activity five times a week (n = 3,846, 83.2%), whereas the least recognized was having a diet low in fiber (n = 1,985, 42.9%; Table 2). This was found in the responses of participants from both the WBJ and the Gaza Strip. The most recognized nonmodifiable CRC risk factor was having a bowel disease (n = 3,320, 71.8%), whereas the least recognized was having diabetes (n = 1,581, 34.2%). This was also noticed in both the WBJ and the Gaza Strip.

**TABLE 2:**
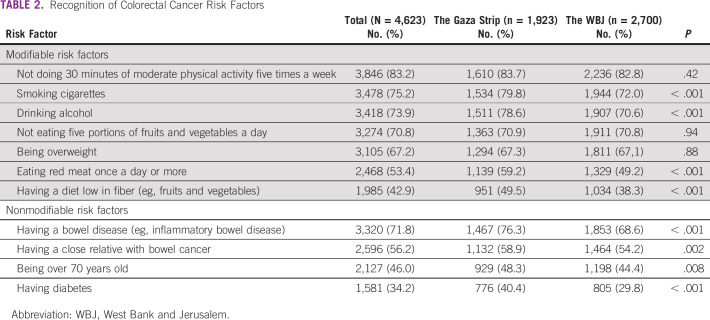
Recognition of Colorectal Cancer Risk Factors

### Good Awareness and Its Associated Factors

A total of 1,840 participants (39.8%) displayed good awareness of CRC risk factors (Table 3). Participants from the Gaza Strip were more likely than participants from the WBJ to have a good level of awareness (46.5.0% *v* 35.0%). The multivariable analysis showed that postsecondary education and knowing someone with cancer were associated with an increase in the likelihood of having good awareness of CRC risk factors (Table 4). Conversely, male sex and living in the WBJ were associated with a decrease in the likelihood of having good awareness.

**TABLE 3:**
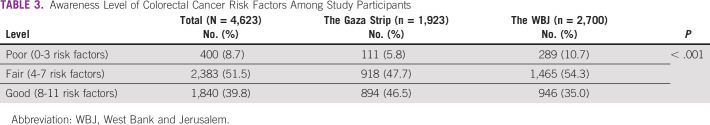
Awareness Level of Colorectal Cancer Risk Factors Among Study Participants

**TABLE 4:**
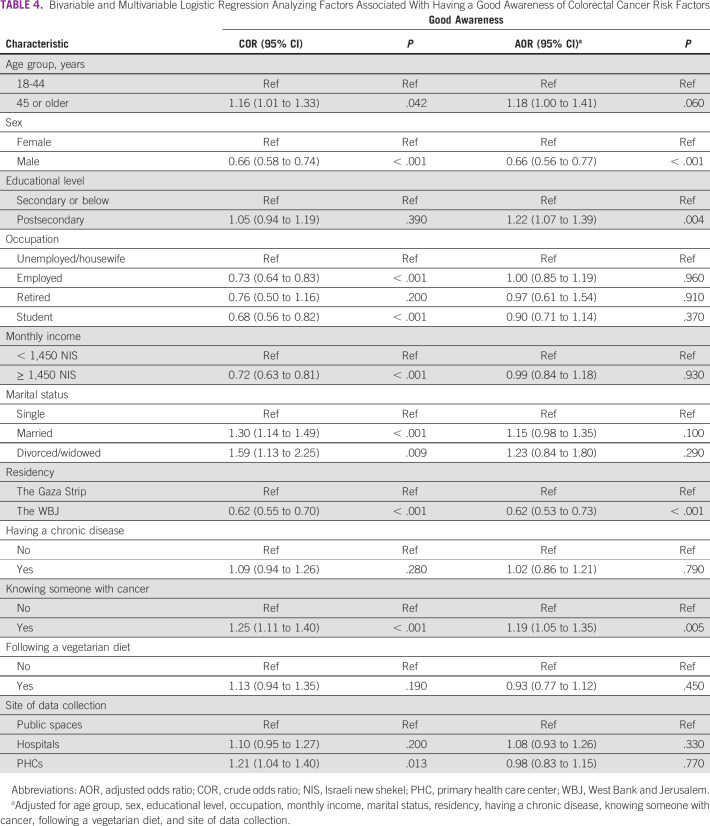
Bivariable and Multivariable Logistic Regression Analyzing Factors Associated With Having a Good Awareness of Colorectal Cancer Risk Factors

### Association Between Recognizing Modifiable CRC Risk Factors and Participant Characteristics

Male participants were less likely than female participants to recognize four of seven modifiable CRC risk factors (Table 5). In addition, participants from the WBJ were less likely than participants from the Gaza Strip to recognize four of the seven modifiable CRC risk factors.

**TABLE 5:**
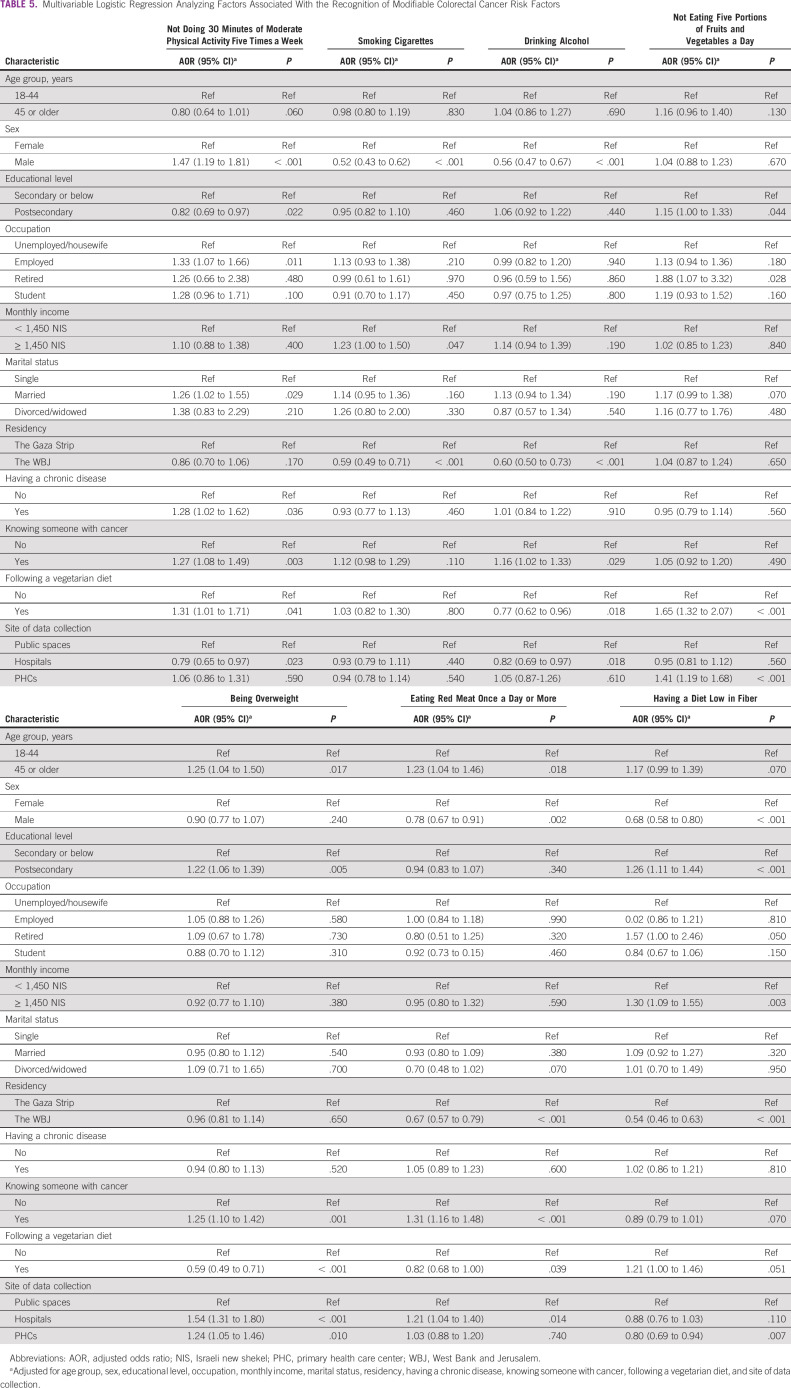
Multivariable Logistic Regression Analyzing Factors Associated With the Recognition of Modifiable Colorectal Cancer Risk Factors

On the other hand, participants who knew someone with cancer were more likely than those who did not to recognize four of the seven modifiable CRC risk factors. Moreover, participants with postsecondary education were more likely to recognize three of the seven modifiable CRC risk factors.

### Association Between Recognizing Nonmodifiable CRC Risk Factors and Participant Characteristics

Participants from the WBJ were less likely than participants from the Gaza Strip to recognize all nonmodifiable CRC risk factors (Table 6). Male participants were less likely than female participants to recognize having a bowel disease (odds ratio [OR] = 0.70; 95% CI, 0.59 to 0.84) and having a close relative with bowel cancer (OR = 0.53; 95% CI, 0.45 to 0.62) as CRC risk factors.

**TABLE 6:**
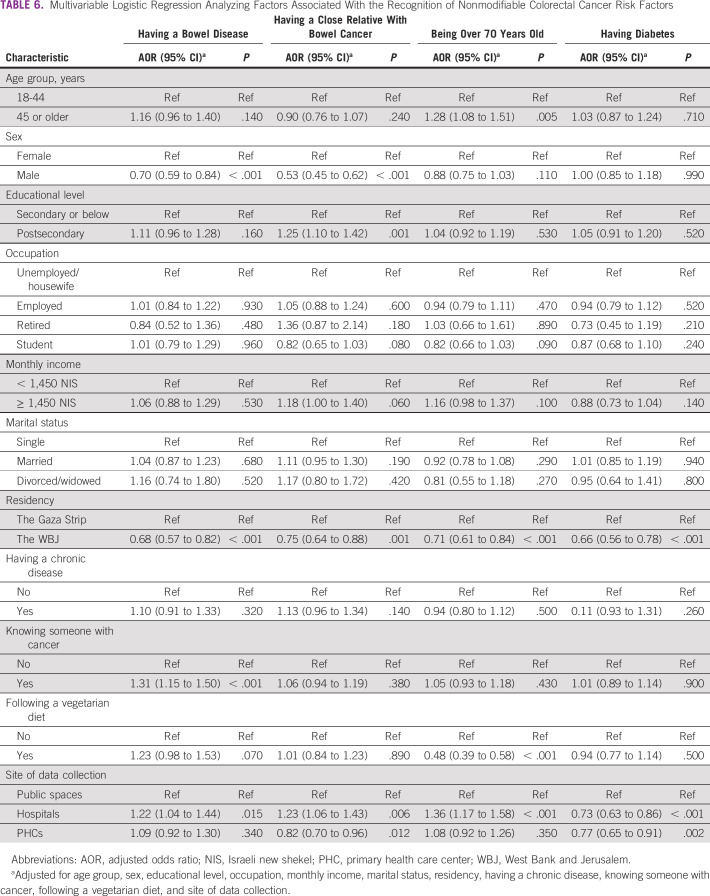
Multivariable Logistic Regression Analyzing Factors Associated With the Recognition of Nonmodifiable Colorectal Cancer Risk Factors

On the contrary, participants recruited from hospitals were more likely than participants recruited from public spaces to recognize all nonmodifiable CRC risk factors except having diabetes for which the opposite was found. Participants eligible for CRC screening (age ≥ 45 years) were more likely to recognize being over 70 years old (OR = 1.28; 95% CI, 1.08 to 1.51) as a CRC risk factor.

## DISCUSSION

A previous study showed that CRC risk factors related to lifestyle behaviors were responsible for 50% of CRC cases in the United Kingdom,^28^ whereas another study from the United States demonstrated that 20%-40% of cancer cases and 50% of cancer-related deaths could be prevented by healthy lifestyle choices.^29^ The main predictors of survival among patients with CRC include advanced stage and late presentation.^7^ Survival rates of CRC can be increased up to 90% if diagnosed at an early stage.^30^ CRC screening has been shown to improve patient outcomes.^5^ However, patients with low CRC awareness were found to be less likely to undergo CRC screening.^8^ The contribution of CRC risk factor awareness to these behavioral changes and practices highlights the need for good public awareness, especially in low- and middle-income countries, such as Palestine, where no CRC screening program exists. This study provides baseline information on the existing awareness about CRC risk factors in Palestine, a low-resource setting, to facilitate future improvements through educational interventions.

In concordance with previous studies from Iran, the United Arab Emirates, Hungary, and the United Kingdom, this study found low awareness of CRC risk factors.^19,31-33^ By contrast, studies from Turkey, Norway, the United States, and Spain found better awareness levels than this study.^18,34-36^ Possible contributing factor to the higher awareness in these studies could be the availability of established national screening programs in these countries, which has been shown to motivate participants to adopt healthier behaviors including those related to CRC.^37,38^ To date, there is no national screening program for CRC in Palestine, and this warrants future consideration to reduce CRC-related mortality.

The good recognition of low physical activity as a modifiable CRC risk factor might be the result of several campaigns conducted in Palestine to promote physical activity among the Palestinian population.^39,40^ Conversely, having a diet low in fibers was the least recognized modifiable risk factor despite the fact that this region (Mediterranean and West Asia) is known for its fiber-rich diet, which has been reported to reduce the risk of developing CRC.^41^ The westernization of the diet in the region over recent years might be an expression of this low awareness of the potential impact of diet on various diseases.^42^ In fact, changes in diet have led to increased incidences of obesity in many low- and middle-income countries; urban and rural populations in the Middle East, sub-Saharan Africa, and South Asia, from the poorest to the wealthiest, appear to have witnessed rapid increases in obesity and overweight.^43^ Despite several diabetes campaigns in Palestine,^44^ having diabetes was the least recognized nonmodifiable risk factor, as also in Qatar, Ethiopia, and the United Kingdom.^12,45-47^ Therefore, awareness campaigns should focus more on the long-term consequences of diabetes including the potential to develop CRC.

The higher level of awareness among women in this study is consistent with previous studies.^10,45,48^ This might be attributed to the fact that women use health care services more often (eg, for maternity care) than men and, thus, come in contact with health care providers more frequently. Therefore, women might have more opportunities to gain information from health care providers on health-related topics, including CRC, which might motivate them to adopt more protective behaviors than men.^49^

Furthermore, this study, as previous studies,^19,50^ found that participants with higher education were more likely to display better awareness. Therefore, future CRC awareness campaigns should target people with lower education.

This study highlights the importance of establishing continuous educational interventions to raise the awareness about risk factors and signs and symptoms of CRC in Palestine. These interventions should focus on different aspects of CRC including its relationship with some chronic diseases (eg, diabetes) and with different diets (eg, fiber-low) as the public awareness about these important risk factors was found to be low. Raising awareness of CRC risk factors may enhance early presentation and, hence, early detection of CRC. Moreover, good awareness of CRC may facilitate the potential implementation of CRC screening as part of future health policy.

The use of convenience sampling limits the generalizability of the findings. However, the large number of participants, the high response rate, and the recruitment from different geographical areas may mitigate this. Furthermore, the exclusion of visitors or patients in oncology departments and participants with medical backgrounds might have reduced the number of participants with a presumably good awareness. Nonetheless, their exclusion was intended to make this study more relevant as a measure of the public awareness.

In conclusion, the awareness level of CRC risk factors was relatively low, with only 39.8% of participants showing good awareness. In general, participants from the Gaza Strip had a higher awareness level than those from the WBJ. The factors associated with having good awareness of CRC risk factors were having postsecondary education and knowing someone with cancer. The most recognized CRC risk factor was not doing 30 minutes of moderate physical activity five times a week, whereas the least recognized was having diabetes. Campaigns and programs aiming to increase awareness of CRC risk factors are needed and should be tailored to address the knowledge gaps among the public.

## Figures and Tables

**TABLE A1:**
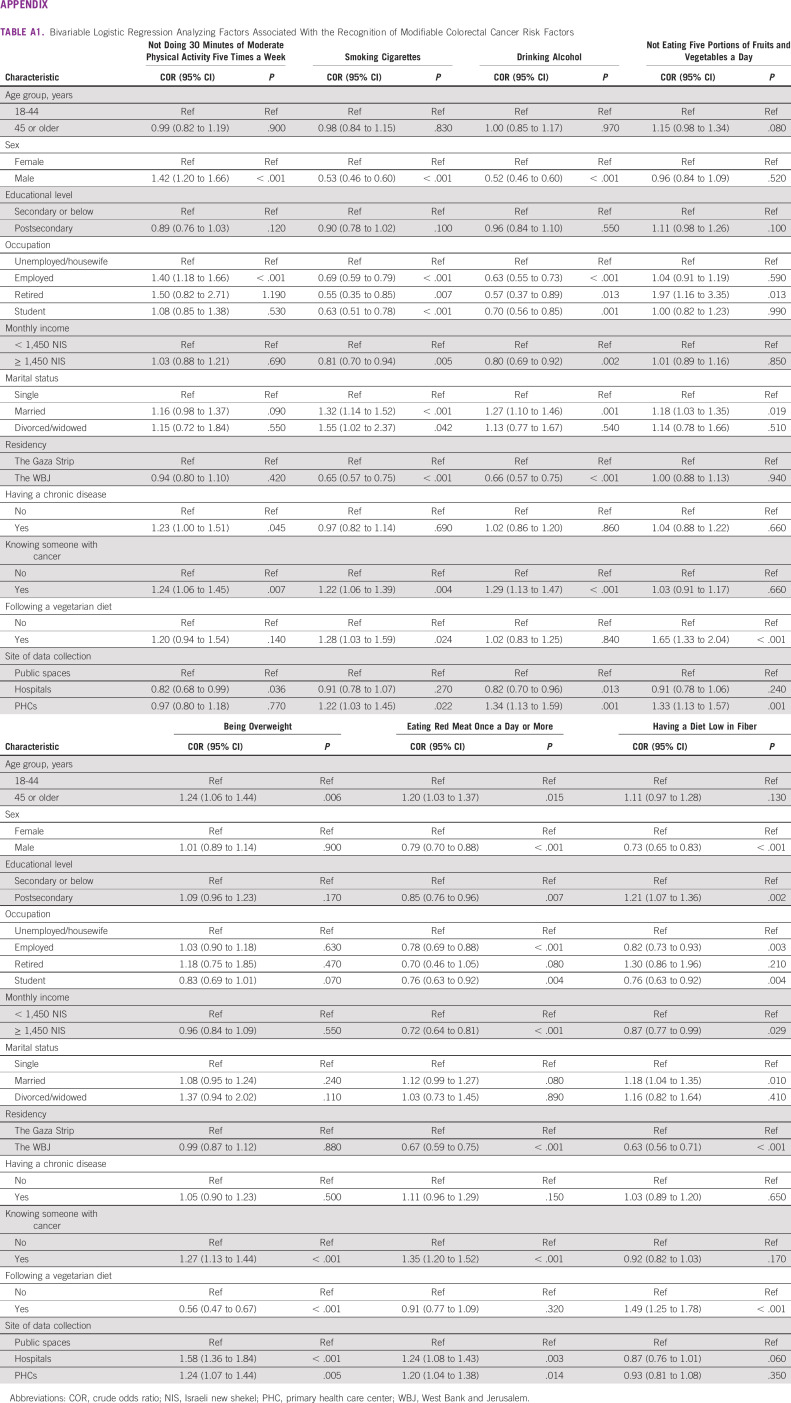
Bivariable Logistic Regression Analyzing Factors Associated With the Recognition of Modifiable Colorectal Cancer Risk Factors

**TABLE A2:**
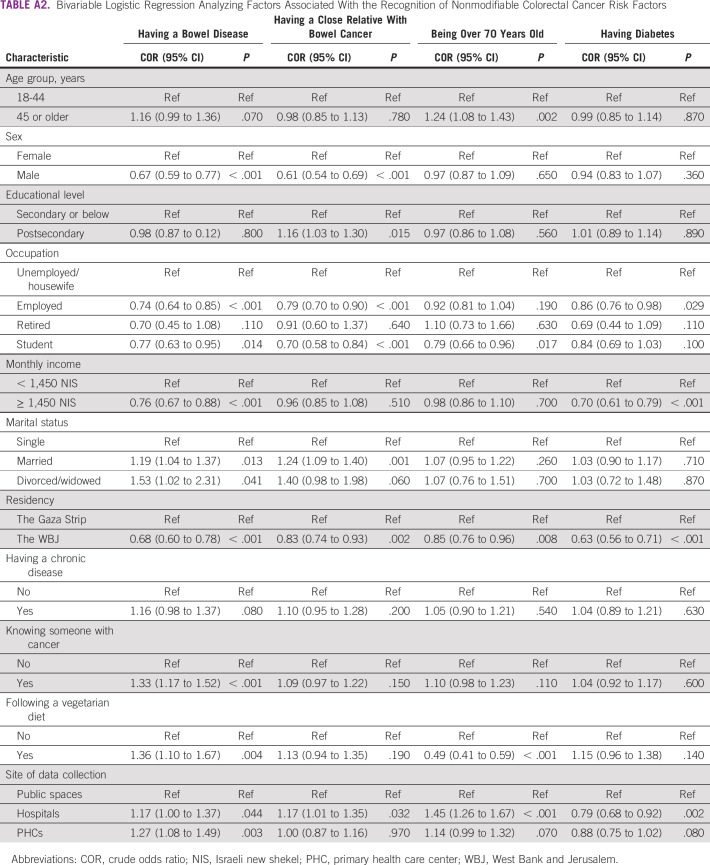
Bivariable Logistic Regression Analyzing Factors Associated With the Recognition of Nonmodifiable Colorectal Cancer Risk Factors
